# Protocol: An updated integrated methodology for analysis of metabolites and enzyme activities of ethylene biosynthesis

**DOI:** 10.1186/1746-4811-7-17

**Published:** 2011-06-23

**Authors:** Inge Bulens, Bram Van de Poel, Maarten LATM Hertog, Maurice P De Proft, Annemie H Geeraerd, Bart M Nicolaï

**Affiliations:** 1 Division of Mechatronics, Biostatistics and Sensors (MeBioS), Department of Biosystems (BIOSYST), Katholieke Universiteit Leuven, Willem de Croylaan 42, bus 2428, B-3001 Leuven, Belgium; 2 Division of Crop Biotechnics, Department of Biosystems (BIOSYST), Katholieke, Universiteit Leuven, Willem de Croylaan 42, bus 2427, B-3001 Leuven, Belgium

**Keywords:** Ethylene, ACC, ACS, ACO, MACC

## Abstract

**Background:**

The foundations for ethylene research were laid many years ago by researchers such as Lizada, Yang and Hoffman. Nowadays, most of the methods developed by them are still being used. Technological developments since then have led to small but significant improvements, contributing to a more efficient workflow. Despite this, many of these improvements have never been properly documented.

**Results:**

This article provides an updated, integrated set of protocols suitable for the assembly of a complete picture of ethylene biosynthesis, including the measurement of ethylene itself. The original protocols for the metabolites 1-aminocyclopropane-1-carboxylic acid and 1-(malonylamino)cyclopropane-1-carboxylic acid have been updated and downscaled, while protocols to determine *in vitro *activities of the key enzymes 1-aminocyclopropane-1-carboxylate synthase and 1-aminocyclopropane-1-carboxylate oxidase have been optimised for efficiency, repeatability and accuracy. All the protocols described were optimised for apple fruit, but have been proven to be suitable for the analysis of tomato fruit as well.

**Conclusions:**

This work collates an integrated set of detailed protocols for the measurement of components of the ethylene biosynthetic pathway, starting from well-established methods. These protocols have been optimised for smaller sample volumes, increased efficiency, repeatability and accuracy. The detailed protocol allows other scientists to rapidly implement these methods in their own laboratories in a consistent and efficient way.

## Background

**Figure 1:**
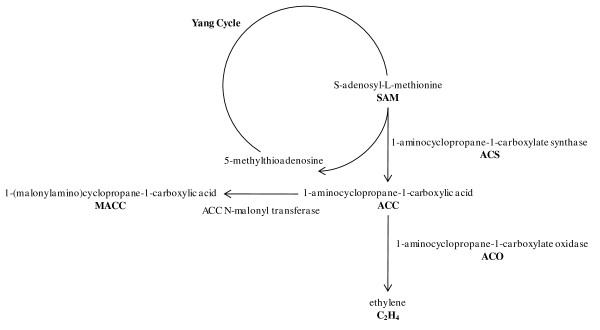
**Pathway of the ethylene biosynthesis**. The ethylene biosynthesis starts from the conversion of S-adenosyl-L-methione (SAM), into 1-aminocyclopropane-1-carboxylic acid (ACC) by the enzyme 1-aminocyclopropane-1-carboxylate synthase (ACS). ACC can then be converted to 1-(malonylamino)cyclopropane-1-carboxylic acid (MACC), by ACC ***N***-Malonyl transferase, or to the end product ethylene, by 1-aminocyclopropane-1-carboxylate oxidase (ACO).

Ethylene biosynthesis starts from the conversion of S-adenosyl-L-methione (SAM) into 1-aminocyclopropane-1-carboxylic acid (ACC) by the enzyme 1-aminocyclopropane-1-carboxylate synthase (ACS). ACC can then be converted to either 1-(malonylamino)cyclopropane-1-carboxylic acid (MACC) by ACC *N*-Malonyl transferase, or to the end product, ethylene, by 1-aminocyclopropane-1-carboxylate oxidase (ACO) [1] (Figure 1).

Analytical protocols for each of these metabolites and enzymes have been developed and are available. The easiest component to measure is the gaseous hormone ethylene. It is formed inside cells, diffuses through the tissue and eventually out of the fruit into the surrounding atmosphere. Therefore multiple readings can be made without destructive sampling of the tissue. Typically, measurements are performed by gas chromatography (GC) [2,3] or more recently by techniques such as photo acoustic laser spectrophotometry (PALS) [4]. Both techniques are easy to use. PALS generally offers a higher detection sensitivity (ppt level; 1:10^12^) compared to GC (ppb level; 1:10^9^). However, GC equipment can be used for a broader range of applications while laser based sensors are highly selective for a specific compound.

In 1979, Lizada and Yang published a method to quantify ACC indirectly by liberating ethylene from ACC with NaOCl in the presence of Hg^2+^[5]. Since then, several methods have been published that allow the direct quantification of ACC by gas chromatography-mass spectrometry (GC-MS) [6], liquid chromatography-mass spectrometry (LC-MS) [7] or capillary electrophoresis with laser-induced fluorescence detection (CE-LIF) [8]. Each technique has its own advantages and disadvantages. For GC-MS and CE-LIF analysis samples need to be derivatised before measurement, which makes the procedure quite complex and time-consuming. The main disadvantage however is the poor reproducibility of the derivatisation procedure when dealing with low ACC concentrations. ACC contents can be directly measured using LC-MS, but the technique is very expensive, both in terms of the equipment required as well as the consumables. This makes LC-MS unfavourable when large amounts of samples are involved. Even though the procedure of Lizada and Yang measures ACC indirectly, it allows for relatively fast measurements with little sample preparation and using low cost equipment. If the fluctuating efficiency of the turnover reaction is taken into account in a proper way, following the protocol outlined in the present manuscript, the technique provides a convenient and accurate way to quantify ACC in, for example, fruit samples.

Hoffman and Yang discovered in 1982 that ACC is not only converted into ethylene but that a large amount of the formed ACC is converted into MACC [9]. They quantified MACC by subtracting the amount of ACC present, prior to and following acidic hydrolysis of the extract, according to the method of Lizada and Yang. The protocol outlined here is based on this original method and provides a rapid procedure to quantify MACC starting from small amounts of ACC extract.

For the quantification of *in vitro *ACS and ACO activity, the protocols presented here are based on more recent work of Vilaplana [10] and Dandekar [11]. The optimisation of these protocols has focussed on volume reductions, the elimination of superfluous handlings and an increased efficiency. Changes have been also made to improve the reproducibility and accuracy of these measurements.

SAM is a less well-studied ethylene precursor, mainly because it is more difficult to quantify [12]. The influence of SAM on ethylene biosynthesis is therefore not known and very often neglected. Recently a paper was published that describes a rapid procedure to quantify SAM by CE-UV [13]. This protocol is compatible with the other protocols described below.

## Results

### Protocol 1 - Ethylene measurement

The measurement of ethylene in the headspace surrounding a sample is quite straightforward, nevertheless it is difficult to directly compare results obtained from different laboratories. For example, when ethylene production is expressed in terms of volume of gas produced, without additional information on the temperature and pressure present during the experimental setup, it cannot be unambiguously related to results expressed in molar units. Ethylene concentrations are quantified by GC. Calibration is done by defining the linear relation between peak area and the known ethylene concentration (ppm) of at least three different calibration concentrations covering the entire detector response range. Before each set of measurements the calibration curve needs to be validated again.

#### Ethylene production rate of intact fruits

1. Fruits are individually enclosed in air tight jars of 1.7 L and flushed for a defined number of hours with humidified air of the desired gas composition until steady state conditions are reached between the headspace and the fruit's internal atmosphere.

2. After flushing, the inlet and outlet of the jar are closed. Before sampling, the pressure inside the jar is measured and subsequently an initial sample of the headspace is taken for GC analysis, using the sampling port of the jar. Here, a total sample volume of 10 mL is extracted to allow proper flushing of the equipment tubing between subsequent samples. After sampling, the pressure inside the jar is again measured. The pressure readings are used to check whether a correct sample is taken, whether the jar is airtight, and to be able to express the amount of ethylene present in molar units using the ideal gas law (*p.V *= *n.R.T*).

3. After an experimentally determined period of time, a second measurement of the headspace is performed, as described in step 2. The length of the period between the two measurements depends on the rate of ethylene production by the sample as the accumulation of ethylene concentrations in the jar has to be sufficiently high to be detectable, but not too high to influence behaviour of the samples. In the case of apples, this was determined to be between 4 and 14 h depending on the ripening stage and the type of experiment. NOTE: *Shorter accumulation periods can also be reached by decreasing the ratio free headspace to total volume of the jar used*.

4. Based on the difference between the final ethylene reading *Eth*_c, f _(ppm) and the initial reading *Eth*_c, i _(ppm), and taking into account the weight of the apple *w *(kg), the free volume of the jar *V*_free _(m^3^), the temperature *T *(K) and the pressure after the first measurement *p*_i _(Pa) and before the second measurement *p*_f _(Pa), the ethylene production rate *Eth*_pr _(mol.kg^-1^.h^-1^) is calculated as follows:(1)(2)(3)

With *n*_eth, f _and *n*_eth, i _being the ethylene concentration (mol), *n*_t _being the total amount of gas in the recipient (mol), *t *being the time between the two readings (h) and *R *the universal gas constant (8.314 J.mol^-1^.K^-1^).

#### Ethylene production for ACC and ACO assays

As will be described in the protocols for the quantification of ACC and the *in vitro *activity of ACO, ethylene production in these assays is measured in a 20 mL glass GC-vial. Due to the small headspace volume an adapted GC method is used where only a small volume of headspace is extracted (3 mL). For the same reason no initial reading is taken and the initial concentration of ethylene in the vial is assumed to be zero.

Again, pressure is measured before and after sampling to control air tightness and ensure correct sampling. Ethylene production is then expressed in moles taking into account temperature, pressure and headspace volume as described in equations 1 and 2 with *n*_eth, i _being zero.

### Protocol 2 - Quantification of ACC and MACC

The two extraction solutions generally used for ACC (and MACC) are sulfosalicylic acid (SSA) and ethanol. In terms of yield, both extraction solutions give similar results, but the repeatability for the SSA extraction is much higher than for the ethanol extraction (data not shown). Another advantage of the SSA extraction is that it is a cold extraction not requiring an evaporation step. This makes the extraction procedure much faster and less laborious. Tests showed that longer extraction times, multiple extraction steps and filtration of the extracts are redundant.

The conversion efficiency of ACC to ethylene in the ACC extracts is matrix, and thereby sample, dependent. For this reason it is essential to measure for each extract an additional spiked sample (with a known amount of ACC added) to determine the reaction efficiency.

#### Preparation of the solutions

1. SSA (5% m/v): Dissolve 5 g of sulfosalicylic acid in a total volume of 100 mL distilled water.

2. HgCl_2 _(10 mM): Dissolve 0.271 g of mercury chloride in a total volume of 100 mL distilled water. NOTE: *This compound is toxic, be sure to read and follow the safety and disposal guidelines provided by the manufacturer with care*.

3. NaOCl (5% v/v): Add 5 mL of sodium hypochlorite to 95 mL distilled water.

4. NaOH (6 M): Dissolve 24 g of sodium hydroxide in a total volume of 100 mL distilled water.

5. HCl (6 M): Dilute a 37% solution of hydrogen chloride to 22% by slowly adding 60 mL of the solution to 40 mL of distilled water.

6. NaOCl-NaOH mixture (2:1, v/v): Add two units of NaOCl to one unit of NaOH and keep on ice. NOTE: *Prepare a fresh NaOCl-NaOH mixture every day*.

7. ACC (50 μM): Make a stock solution of 10 mM by dissolving 2 mg of ACC in 2 mL distilled water. Dillute the stock solution to 50 μM. NOTE: *Store the ACC solution at -20°C*.

#### Extraction

See Figure 2

**Figure 2:**
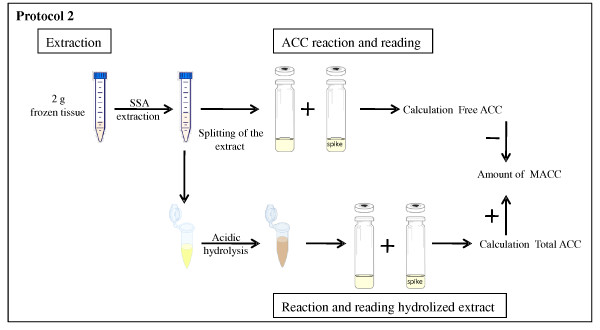
**Schematic overview of the ACC and MACC protocol (protocol 2)**. Following extraction of tissue with SSA, the extract is divided into two aliquots. The first aliquot is used for the determination of the free ACC content of the sample, while the second is first hydrolysed, before the total ACC content of the sample is measured. The MACC concentration of the sample is calculated by subtracting the free ACC concentration from the total ACC concentration.

1. Weigh 2 g of crushed, frozen tissue in a 15 mL falcon tube cooled in liquid nitrogen. Store in liquid nitrogen or at -80°C until extraction.

2. Add 4 mL of 5% SSA solution to the frozen sample. Vortex until a homogeneous mixture is obtained.

3. Allow 30 min for the extraction to take place while gently shaking the sample at 4°C.

4. Centrifuge the sample for 10 min at 3,090 × g in a precooled centrifuge at 4°C. Collect the supernatant (around 4.5 to 5 mL) in a clean 15 mL falcon.

5. Separate 0.5 mL of the collected supernatant in a 1.5 mL microcentrifuge tube for acidic hydrolysis to liberate MACC.

6. Store both the falcon and the microcentrifuge tube in liquid nitrogen or at -80°C until performing the next steps of the protocol.

#### Acidic hydrolysis

1. Heat the water bath to 99°C.

2. Thaw the microcentrifuge tube containing 0.5 mL of ACC extract and add 0.2 mL of 6 M HCl. Vortex the sample to homogenise the solution.

3. Put the microcentrifuge tube in the water bath (using floating holders) and allow 3 h for the reaction. NOTE: *Shortly after placing the microcentrifuge tube in the water bath it might pop open due to the sudden change in temperature. It is recommended to use safe lock microcentrifuge tubes to avoid popping of the tubes*.

4. After 3 h, remove the microcentrifuge tubes from the water bath and allow them to cool down for 15 min at room temperature. Add 0.2 mL of 6 M NaOH and vortex briefly. NOTE: *This step is performed to neutralize the sample and results in a dark brown solution*.

5. Centrifuge the microcentrifuge tube for 5 min at 22,000 × g. Discard the brown pellet and collect the yellow supernatant.

6. Store the supernatant at -20°C until performing the reaction step.

#### Reaction and reading of the ACC extract

Because the efficiency of the reaction to turn ACC into ethylene is sample dependent, each sample should be analysed twice. In the second measurement, a known amount of ACC is added to the extract (step 3 in de reaction protocol described below). Based on the results of both reactions, the efficiency which is used to correct the final outcome is then determined for each individual sample.

1. Thaw the sample on ice.

2. Bring 1.4 mL of the ACC extract in a glass vial (e.g. 20 mL).

3. If necessary spike the sample by adding 20 μL of a 50 μM ACC solution. NOTE: *The amount of ACC added by spiking should be in the similar concentration range as the natural occurring ACC content of the sample itself*.

4. Add 0.4 mL of 10 mM HgCl_2 _and immediately seal the vial airtight with a septum containing cap.

5. Inject 0.2 mL of the NaOH-NaOCl mixture through the septum of the cap using a needle and syringe. NOTE: *The turnover of the reaction is highly dependent on the amount of NaOH-NaOCl mixture added. So it is very important to add the required amount as accurately as possible*.

6. Vortex the sample for 5 seconds and allow it to react for 4 min on melting ice.

7. Vortex the sample again for 5 seconds to release all ethylene into the vial headspace. NOTE: *The reaction time and the time taken to vortex must be controlled with great care to minimise errors*.

8. Immediately take a sample of the headspace for GC analysis of the content. NOTE: *During the 4 min reaction time another sample can be prepared. Ensure that the time between sample processing is sufficient to perform the GC analysis*. NOTE: *Each time a new ACC solution to spike is prepared, three blank spiked samples should be tested (by replacing extract with water) to calibrate the concentration of the spike. We assume 100% reaction efficiency in water*.

#### Reaction and reading of the hydrolysed ACC extract

Except for the volumes added, this reaction is identical to the one for the ACC extract, with again every sample being analysed twice (either spiked or not with a known amount of ACC). Instead of the 1.4 mL of extract (step 2) use 0.6 mL distilled water, with 0.1 mL hydrolysed extract. Instead of the 0.4 mL HgCl_2 _(step 4) add 0.2 mL HgCl_2_. During step 5, after sealing the vial, 0.1 mL of the NaOH-NaOCl mixture is injected through the septum of the cap.

NOTE: *The reduction in the amount of extract and the addition of water, compared to the reaction of the ACC extract, is done to increase the reaction efficiency to about 70%. Adding more extract (containing more ACC) will only lead to a higher ethylene production if the efficiency of the reaction is kept high enough. This is done by diluting the extract with water, and by proportionally increasing the amount of HgCl_2 _and NaOH-NaOCl mixture*.

NOTE: *The blank spike value has to be determined separately for the hydrolysed samples because it will differ slightly with the one for the non hydrolysed extracts due to small changes in the amount of added products*.

#### Calculation of ACC and MACC concentration

Based on the GC readings of the sample *Eth*_sample _and the spiked sample *Eth*_spike _(ppm), the number of moles ethylene formed, *n*_sample _and *n*_spike _can be calculated as follows:(4)(5)

with *V*_free _being the volume of the GC vial minus the total amount of liquids added (m³), *p*_i _the pressure before sample taking, *T *the temperature (273 K) and *R *the universal gas constant (8.314 J.mol^-1^.K^-1^).

For each sample the reaction efficiency (*Eff*) of the ACC turnover can be calculated as:(6)

with n_blank _being the average of the amount of moles ethylene formed in the three blank samples containing only spiking solution.

Taking into account the amount of extract used for the reading *V*_reading_, the total volume of liquid in the sample after extraction (considering the water content of the tissue) *V*_extract _and the weight of the used sample *w*, the amount of *ACC *in the sample (mol.g FW^-1^) equals:(7)

The amount of MACC in the sample is calculated by subtracting the free ACC concentration (*ACC*_free_) from the hydrolysed ACC concentration (*ACC*_tot_):(8)

### Protocol 3 - *In vitro *activity of ACS

The quantification of *in vitro *ACC synthase activity is the most time consuming and extensive protocol since it requires enzyme extraction, cleaning up of the extract (to discard endogenous ACC and other interfering compounds), and a two step reaction to convert SAM via ACC into ethylene.

Compared to the original protocol, less starting material is needed and time consuming steps, such as filtering through cheesecloth, were shown to be redundant. Tests indicated that it is possible to store extracts and reacted samples at -80°C without affecting the measured activity or influencing any further steps. This allows for a more time efficient approach since different parts of the protocol can now be performed over different days with an optimised number of samples.

**Figure 3:**
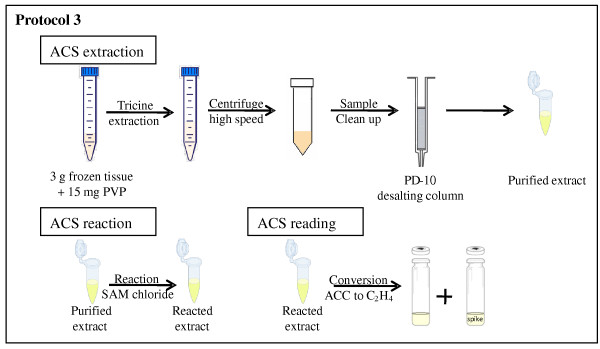
**Schematic overview of the ACS protocol (protocol 3)**. Following tricine extraction of the tissue, a sample clean up on a PD-10 desalting column is performed to remove salts and ACC from the extract. After reaction of the purified extract with SAM chloride, the amount of ACC formed is quantified by chemically converting it into ethylene.

A schematic overview of the protocol is given in Figure 3.

#### Preparation of the solutions

1. Tricine extraction buffer (200 mM, pH 8.5): Dissolve 8.96 g of Tricine in distilled water, adjust to pH 8.5, add distilled water up to a total volume of 250 mL.

2. Tricine reaction buffer (200 mM, pH 8.0): Dissolve 8.96 g of Tricine in distilled water, adjust to pH 8.0, add distilled water up to a total volume of 250 mL.

3. Tricine column buffer (5 mM, pH 8.0): Dissolve 0.224 g of Tricine in distilled water, adjust to pH 8.0, and add distilled water up to a total volume of 250 mL.

4. PLP (2 mM): Dissolve 2.47 mg of pyrodoxal-L-phosphate (PLP) in a total volume of 5 mL distilled water.

5. Extraction solution: Add 150 μl PLP (2 mM) and 115.5 mg of DTT (10 mM) to 75 mL of Tricine extraction buffer (200 mM, pH 8.5)

6. Column solution: Add 405 μl PLP (2 mM) and 62.6 mg of DTT (1 mM) to 405 mL of Tricine column buffer (5 mM, pH 8).

NOTE: *Prepare PLP and consequently all extraction and column solutions fresh daily*.

7. HgCl_2 _(100 mM): Dissolve 2.71 g of mercury chloride in a total volume of 100 mL of distilled water.

8. NaOCl-NaOH mixture (2:1, v/v): prepare as described for the ACC protocol.

9. SAM chloride (1.2 mM): Dissolve 13 mg of SAM chloride in a total volume of 25 mL distilled water. Vortex until all powder is dissolved, then divide into 20 aliquots of 1.25 mL using microcentrifuge tubes and store at -80°C.

NOTE: *Be sure to use SAM chloride and not SAM iodide; with SAM iodide ACC synthase does not seem to react*.

#### Extraction and purification

1. Weigh 15 mg of polyvinylpyrrolidone (PVP) in a 15 mL falcon.

2. Add 3 g of frozen crushed sample to the tube containing PVP after cooling the falcon in liquid nitrogen.

3. Add 3 mL of extraction solution and vortex until a homogeneous mixture is obtained.

NOTE: *It is important the keep the sample on ice to avoid loss of activity*.

4. Centrifuge the sample for 20 min at 24,414 × g at 4 °C (cool rotor in advance). Collect 3 mL of the supernatant in a cooled 15 mL falcon.

5. Load 2.5 mL of the supernatant on a Sephadex G-25 desalting column for sample clean up. Elute the sample from the column with 3.5 mL of the same column solution and collect this fraction in a cooled falcon (keep on ice). NOTE: *The columns are disposable but can be reused about four times. They should be discarded when flow through becomes very slow. The columns can be stored at 4°C after adding 2 mL of column solution to prevent drying out*. *Equilibrate the columns before every use with 10 mL of column solution*.

6. Transfer 1.5 mL of the eluate in two separate 2 mL microcentrifuge tubes (one of them to be used for the reaction, the other as a spare). Immediately snap freeze in liquid nitrogen to avoid loss of activity. The purified extract can be stored at -80°C until reaction is performed.

#### Reaction

1. Thaw the purified extract on ice.

2. Add 150 μL of Tricine reaction buffer and 150 μL of SAM chloride to the 1.5 mL extract, vortex briefly.

3. Allow the sample to incubate in a thermo mixer for 2 h at 25°C while gently shaking. The ACC synthase will now convert part of the excess SAM substrate into ACC.

4. After 2 h, remove samples and stop the reaction by adding 200 μL of the 100 mM HgCl_2 _solution. NOTE: *The high concentration of HgCl_2 _stops any enzyme activity*.

5. Keep samples on ice if the readings steps take place straight away, otherwise snap freeze in liquid nitrogen and store at -80°C.

#### Reading

Except for the fact that HgCl_2 _has already been added to the sample and for the slightly different volumes of reactants added, this reaction is identical to the ones for ACC and the hydrolysed ACC extract. Again every sample is analysed twice (either spiked or not spiked with a known amount of ACC).

Put 850 μL distilled water in a 20 mL glass vial and add 950 μL of the reacted extract (in case of the spiked sample add 20 μL of a 50 μM ACC solution). Seal the vial with a cap and inject 0.2 mL of the NaOH-NaOCl mixture through the septum of the cap. Follow steps 6-8 in "Reaction and reading of the ACC extract" protocol 2.

#### Calculation of the *in vitro *activity of ACS

The calculation of the amount of moles ethylene formed in the sample *n*_sample _and of the efficiency *Eff *is described in protocol 2 by equations 4, 5 and 6. Taking into account the amount of extract used in the reading *V*_reading_, the volume of the liquid in the sample after extraction (considering the water content of the tissue) *V*_extract_, the dilution factor due to sample clean up on the column *D*_column_, the dilution factor due to the reaction step *D*_reaction_, the weight of the used sample *w *(g FW) and the incubation time *t *(h), ACS activity in the sample (mol.g FW^-1^.h^-1^) equals:(9)

with *D*_column _= 1.4 and *D*_reaction _= 1.33.

### Protocol 4 - *In vitro *activity of ACO

**Figure 4:**
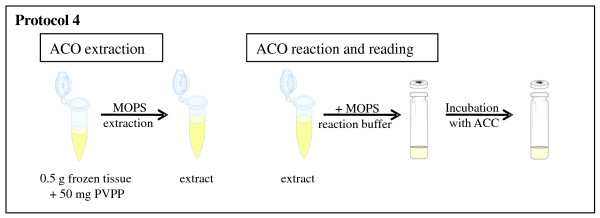
**Schematic overview of the ACO protocol (protocol 4)**. Following extraction of tissues, MOPS reaction buffer and an excessive amount of ACC are added to the extract. After incubation at 30°C for 1 h the amount of ethylene formed is measured by GC.

In order to quantify the *in vitro *activity of ACO, the extracted enzyme is incubated with an excess of ACC and the ethylene formed is measured (Figure 4).

Again, the emphasis during protocol optimisation was on scale reduction and an increased efficiency. Tests showed that it is possible to perform the extraction and the reaction on different days, as long as the extracts are immediately frozen in liquid nitrogen. When stored on ice there is a slow but consistent loss of activity. Another aspect that was added to the protocol is the importance of a blank reading. The measurement of blank samples is necessary to incorporate the amount of non-enzymatic ethylene production from the reaction buffer.

#### Preparation of the solutions

1. MOPS extraction buffer (400 mM, pH 7.2): Dissolve 8.370 g of MOPS in 50 mL of distilled water. Add 10 mL of glycerol (10% v/v) and 0.594 g of ascorbic acid sodium salt (30 mM). Homogenise the solution, adjust to pH 7.2, and add distilled water to a total volume of 100 mL.

2. MOPS reaction buffer (50 mM, pH 7.2): Dissolve 1.046 g of MOPS in 50 mL of distilled water. Add 10 mL of glycerol (10% v/v), 0.099 g of ascorbic acid sodium salt (5 mM), 0.168 g of sodium bicarbonate (20 mM), 0.3 mg of iron sulphate (0.02 mM), 0.010 g ACC (1 mM) and 0.015 g DTT (1 mM). Homogenise the solution, adjust to pH 7.2, and add distilled water to a total volume of 100 mL.

NOTE: *Since ascorbic acid and DTT are very unstable they need to be added shortly before the buffers are used. Preferably buffers are prepared fresh daily and kept cold on ice*.

NOTE: *For tomato samples better results are obtained when a Tris buffer is used (100 mM, pH 8.0) instead of the described MOPS buffer*.

#### Extraction

1. Weigh 50 mg of polyvinylpolypyrrolidone (PVPP) in a 2 mL microcentrifuge tube. NOTE: *PVPP will catch interfering polyphenols what will result in an increase in the observed ACO enzyme activity*.

2. Add 500 mg of crushed tissue after cooling the microcentrifuge tube in liquid nitrogen.

3. Add 1 mL of extraction buffer. Vortex until a homogeneous mixture is obtained.

4. Allow the sample to incubate in a thermo mixer for 10 min at 4°C while gently shaking.

5. Centrifuge the sample for 30 min at 22,000 × g in a precooled centrifuge at 4°C, discard the pellet and collect the supernatant (around 800 μl, depending on the water content of your sample) in a 1.5 mL microcentrifuge tube.

6. Keep the sample on ice if the reading takes place immediately after. Otherwise snap freeze in liquid nitrogen and store at -80°C.

NOTE: *Be sure to keep the sample cold or on ice during and in between the extraction steps to avoid loss of activity*.

#### Reaction and Reading

1. Thaw the frozen extract on ice.

2. Add 3.6 mL of MOPS reaction buffer together with 400 μl of extract in a 20 mL glass vial, close immediately with a disposable cap.

3. Vortex for 5 seconds to homogenise the mixture.

4. Allow the sample to incubate in a water bath for exactly 1 h at 30°C while gently shaking.

5. Vortex again for 5 seconds, to release all ethylene into the vial headspace. NOTE: *It is very important that vortexing is always done in the same way and for the same duration because it can have a significant influence on the reading of the ethylene concentration*.

6. Immediately take a sample of the headspace with the GC to analyse ethylene levels.

NOTE: *Before and after a series of measurements (for example 30 readings) it is important to measure a blank sample (3.6 mL reaction buffer + 0.4 mL distilled water) since small amounts of ethylene can be formed by the buffer due to non-enzymatic interactions between the reagents. Values obtained for the blank samples typically vary between 15 and 50 ppb ethylene. The amount of ethylene formed by the buffer can change over time. By measuring how the blank value changes over time, a distinction can be made between ethylene production from the buffer itself and ethylene production due to the ACC oxidase activity in the extract*.

#### Calculation of the *in vitro *activity of ACO

The amount of ethylene formed *n*_sample _(mol) taking into account the non-enzymatic formation of ethylene can be calculated as follows:(10)

with *Eth*_sample _and *Eth*_blank _(ppm) being the measurements of the sample and corresponding blank respectively, *p*_i _(Pa) the pressure before measurement, *V*_free _(m³) the free headspace volume in the GC vial, *R *the universal gas constant (8.314 J.mol^-1^.K^-1^) and *T *the temperature (K).

The *in vitro *activity of ACO (mol.g FW^-1^.h^-1^) is expressed as:(11)

with *V*_reading _(mL) the amount of extract used in the reading, *V*_extract _(mL) the volume of the liquid in the sample after extraction, *w *(g FW) the weight of the sample and *t *(h) the incubation time.

## Comments

In this manuscript we provide a detailed and up to date set of protocols to measure all ethylene biosynthetic intermediates based on literature and personal observations. The protocols have been extensively tested for apple and tomato and are a good starting point for any ethylene related research. Table 1 provides an overview of the range of results that can be expected for unripe and ripe apple and tomato fruit, for the various protocols. When working with different products it is advisable to verify the efficiencies of the ACC turnover in protocol 2 and to adjust the ratios of the components in the mixture if necessary. In protocol 4, the incubation time of the ACO extract should be checked and if necessary optimized by measuring the variation in ACO enzyme activity over time. The increase in activity should be linear within the time frame used. It is also important to check the literature for the optimum pH of ACS and ACO, and for possible alternative buffers which might yield a higher activity for other type of samples.

**Table 1:** Overview of the expected range for unripe and ripe apple and tomato fruit

	Apple		Tomato	
	
	Unripe	Ripe	Unripe	Ripe
Ethylene production rate (nmol/kg.s)	0.0001	1.228	0.0014	0.0361

ACS activity (nmol ACC/kg.s)	< 0.004	1.069	0.004	0.017

ACC (nmol/g)	0.08	1.19	0.05	2.55

MACC (nmol/g)	1.32	35.77	1.00	20.00

ACO activity (nmol C_2_H_4_/kg.s)	0.032	1.191	0.222	0.889

All values are an average of 8 repetitions of fruits of the same ripening stage.

## Materials

Table 2 contains an overview of all the chemical products used in the protocols.

**Table 2:** List of chemical components used

Name	Abbreviation	CAS number	MW	Company
1-Aminocyclopropane-1-carboxylic acid	ACC	22059-21-8	101.10	Acros Organics (Geel, Belgium)

3-Morpholinopropane-1-sulfonic acid	MOPS	1132-61-2	209.27	Applichem (VWR, Leuven, Belgium)

Ascorbic acid sodium salt		134-03-2	198.11	Fluka (Sigma-Aldrich, St Louis, MO, USA)

Dithiothreitol	DTT	3483-12-3	154.26	Sigma-Aldrich (St Louis, MO, USA)

Glycerol		56-81-5	92.09	Acros Organics (Geel, Belgium)

Hydrogen chloride (37%)	HCl	7647-01-0	36.46	Acros Organics (Geel, Belgium)

Iron Sulphate		7720-78-7	151.91	Acros Organics (Geel, Belgium)

Mercury chloride	HgCl_2_	7487-94-7	271.52	Acros Organics (Geel, Belgium)

Polyvinylpyrrolidone	PVP	9003-39-8		Applichem (VWR, Leuven, Belgium)

Polyvinylpolypyrrolidone	PVPP	94800-10-9		Applichem (VWR, Leuven, Belgium)

Pyridoxal-L-phosphate	PLP	54-47-7	271.14	Sigma-Aldrich (St Louis, MO, USA)

S-(5-Adenosyl)-L-methionine chloride	SAM chloride	24346-00-7	434.90	Sigma (Sigma-Aldrich, St Louis, MO, USA)

Sodium bicarbonate		144-55-8	84.01	Fluka (Sigma-Aldrich, St Louis, MO, USA)

Sodium hydroxide	NaOH	1310-73-2	40.00	Thermo Fisher Scientific (Waltham, MA, USA)

Sodium hypochlorite	NaOCl	7681-52-9	74.44	Aldrich (Sigma-Aldrich, St Louis, MO, USA)

Sulfosalicylic acid (dihydrate)	SSA	5965-83-3	254.21	Acros Organics (Geel, Belgium)

Tricine		5704-04-1	179.17	Sigma (Sigma-Aldrich, St Louis, MO, USA)

The GC used was a Compact GC (Interscience, Louvain La Neuve, Belgium) equipped with a Porabond Q column (ID 0.53 mm) and FID detector.

In the protocols three different types of centrifuges are used depending on the sample volume and the required speed: Eppendorf Centrifuge 5417 R (Eppendorf, New York, United States), Rotina 48 R (Hettich GmbH, Tuttlingen, Germany) and UniCen 15 DR (Herolab GmbH, Wiesloch, Germany).

The PD-10 desalting columns used in protocol 3 are prepacked disposable columns containing Sephadex™ G-25 Medium (GE Healthcare Europe, Diegem, Belgium).

## List of abbreviations used

ACC: 1-Aminocyclopropane-1-carboxylic acid; ACO: 1-Aminocyclopropane-1-carboxylate oxidase; ACS: 1-Aminocyclopropane-1-carboxylate synthase; CE-LIF: Capillary electrophoresis with laser-induced fluorescence detection; CA: Controlled atmosphere; GC: Gas chromatography; GC-MS: Gas chromatography-mass spectrometry; LC-MS: Liquid chromatography-mass spectrometry; MACC: 1-(Malonylamino)cyclopropane-1-carboxylic acid; PALS: Photo acoustic laser spectrophotometry; SAM: S-Adenosyl-L-methione

## Competing interests

The authors declare that they have no competing interests.

## Authors' contributions

IB and BVdP designed the study and carried out the experimental work. IB drafted the manuscript. MH guided the design of the experiments, the interpretation of the results and the writing of the manuscript. AG and BN contributed to the experimental design and the data interpretation. MDP participated in the optimization of the ACC protocol. BVdP and MH revised the manuscript critically and extensively. All authors read and approved the final manuscript.
